# Key Bacteria in the Gut Microbiota Network for the Transition between Sedentary and Active Lifestyle

**DOI:** 10.3390/microorganisms8050785

**Published:** 2020-05-24

**Authors:** Nazareth Castellanos, Gustavo G. Diez, Carmen Antúnez-Almagro, Carlo Bressa, María Bailén, Rocío González-Soltero, Margarita Pérez, Mar Larrosa

**Affiliations:** 1 Nirakara Lab, Mindfulness and cognitive Science extraordinary Chair, Universidad Complutense de Madrid, 28223 Madrid, Spain; nazareth@nirakara.org (N.C.); gustavo@nirakara.org (G.G.D.); 2 Dementia Care Unit, Virgen de la Arrixaca University Hospital, 30120 Murcia, Spain; mcarmen.antunez@carm.es; 3 Masmicrobiota group, Faculty of Biomedical and Health Sciences, Universidad Europea de Madrid, 28670 Madrid, Spain; carlo.bressa@universidadeuropea.es (C.B.); maria.bailen@universidadeuropea.es (M.B.); mariadelrocio.gonzalez@universidadeuropea.es (R.G.-S.); 4 Faculty of Sport and Health Sciences, Universidad Europea de Madrid, 28670 Madrid, Spain; margarita.perez@universidadeuropea.es

**Keywords:** network topology, microbial interactions, transition bacteria, physical exercise, sedentarism, network flow coefficient, *Roseburia*, *Sutterella*

## Abstract

Physical activity modifies the gut microbiota, exerting health benefits on the host; however, the specific bacteria associated with exercise are not yet known. In this work, we propose a novel method, based on hierarchical topology, to study the differences between the microbiota of active and sedentary lifestyles, and to identify relevant bacterial taxa. Our results show that the microbiota network found in active people has a significantly higher overall efficiency and higher transmissibility rate. We also identified key bacteria in active and sedentary networks that could be involved in the conversion of an active microbial network to a sedentary microbial network and vice versa.

## 1. Introduction

The benefits of performing physical activity on health are well known; the practice of physical exercise improves cardiovascular health, attenuates aging dysfunction, and prevents and improves recovery in cancer [1,2]. On the other hand, sedentary lifestyles have recently emerged as a new risk factor for health. The detrimental effects of a sedentary lifestyle on health are independent of the level of physical activity, as sedentary behavior considerably increases the prevalence of chronic diseases by itself [3]. Moderate exercise for 60–75 min per day is able to reduce the mortality associated with sitting for more than eight hours per day, but does not counter the increased risk associated with high TV viewing [4]. One-third of the adult world’s population does not reach public health guidelines for recommended levels of physical activity [5]. A novel factor through which physical exercise impacts health is gut microbiota. Exercise can promote changes in gut microbiota through several mechanisms, such as the release of myokines, the increase of intestinal transit, or the segregation of hormones and neurotransmitters [6]. In turn, gut microbiota can influence exercise adaptations by influencing energy metabolism, improving the immune response, regulating the redox status, affecting the hydration status, or improving the endurance performance by its fermentation capacity [7]; therefore, knowing how physical exercise influences the microbiota community structure is essential to understanding the relationship between both factors. The equilibrium between microbiota components, estimated by employing network analysis, is a promising approach that allows us to determine which factors influence gut microbiota and how to modulate it. In previous work, we determined how an active lifestyle is associated with a more complex microbiota network, which can lead to a more resistant and resilient microbiota [8]. Based on that work, we analyzed the microbiota to identify the most relevant bacteria in the microbiota network reorganization by estimating the transition process between sedentary and active condition through topological measurements of the networks. The identification of these keystone bacteria associated with exercise could relevant to understanding exercise benefits or predicting the risks associated with a lack of physical activity.

## 2. Materials and Methods

### 2.1. Data Collection

We used data from a previous study [8] to estimate the network topology. The Ethics Committee for Clinical Research of the Ramón y Cajal Hospital CEIC 338-14 (Madrid, Spain) approved the study. All procedures were in accordance with the 1964 Declaration of Helsinki and its later amendments. Written informed consent was obtained from all participants. Raw data, stored in FASTQ file format, were deposited into the National Center for Biotechnology Information (NCBI) Biosample database and is publicly accessible with the accession number PRJNA564612. Briefly, a cohort of 109 volunteers was classified into either the active (ACT) (*n* = 64) or sedentary (SED) (*n* = 45) categories, according to the World Health Organization criteria [9]. Physical activity was characterized by using accelerometers (for details, see [8]).

### 2.2. Microbiota and Network Analysis

Gut microbiota was analyzed from the stool DNA, which was extracted using the commercial EZNA Stool DNA Kit (Omega BioTek, Madrid, Spain) with a bead-beating homogenizer (Bullet BlenderStorm, Next Advance, New York, NY, USA). Microbiota analyses were performed by amplifying the V3–V4 hypervariable regions of the bacterial 16S rRNA gene. DNA amplicons were sequenced on a MiSeq Illumina platform (Illumina, San Diego, CA, USA) and analyzed using the Quantitative Insights into Microbial Ecology (QIIME) program, version 1.9.1 [10] (Available online: http://qiime.org). Gut microbiota components (OTU abundances) were treated as nodes of a network, and estimated by the pairwise Spearman correlation, with a survival statistical criterion of *p* < 0.00001 for multiple comparison correction (for a review, see [11,12], and for details on this dataset, see [8].

The network topological characterization was as follows. (i) Clustering (C) measures the number of connections between the neighbors of a bacterial taxon as the proportion of the maximum number of possible connections. Low values of C code for a small number of clusters in the network; (ii) Efficiency (E) is the average of the shortest path length in the network, measured as the minimum number of nodes that must be traversed to go from one node to another. Low values of E indicate a topological distance between bacterial taxa. (iii) Flow coefficient (FC) measures the capability of a network to transmit or propagate. In terms of nodes, FC is a measure of the betweenness centrality and hence has relevant importance for both topology and biological implications, and was used in this work to estimate the bacteria relevance. The assortativity index measures the trend to link to other bacteria with the same or similar degree. Finally, we define the transition networks as those driving the transition between conditions ACT and SED. Under the hypothesis that the microbiota network in the ACT lifestyle can be reorganized into a SED network when a person abandons an active lifestyle [11,12]. We can represent this transition process as a matrix dot product as follows: SED = Y.ACT, where Y represents the transition network from an ACT to a SED lifestyle, and is estimated as Y = inv(SED).ACT (inv represents the inverse of a matrix). Similarly, for the transition from a SED to an ACT lifestyle, X = inv(ACT).SED. X and Y are networks representing the transition processes and can be analyzed topologically. We estimated the keystone bacteria of X and Y to identify the most relevant bacteria in the transition between conditions. The keystone bacteria are those with the 10% highest values of FC in X and Y (a standard procedure for hubs identification in network analysis).

### 2.3. Statistical Analysis

Statistical analysis was carried out using SPSS software 21.0 (SPSS, Chicago, IL, USA). The normality of distribution (Kolmogorov–Smirnov test) was checked before analyses. The topological values were statistically compared using a Kruskal–Wallis test, where the *p*-value was adjusted using the Bonferroni correction, establishing the critical value of *p* < 0.0001. Networks were first normalized by the parameters obtained after using 1000 surrogates where the distribution of the links was kept intact using a custom-made script in Matlab R2018b (Natick, MA, USA).

## 3. Results

### 3.1. Population Characteristics

A total of 109 healthy individuals participated in the study: 64 in the ACT group and 45 in the SED group. The mean age was 32.17 ± 7.40 years in the ACT group and 33.69 ± 7.96 in the SED group. Body mass index (BMI) was 24.01 ± 3.28 kg/m^2^ for the ACT group and 23.63 ± 2.91 kg/m^2^ for the SED group, with no significant differences between them (*p* = 0.71); however, significant differences (*p* < 0.001) were found between the SED and ACT participants in the following: body fat percentage, ACT = 22.72% and SED = 32.36%; visceral fat mass, ACT = 274.39 g and SED = 417.66 g; muscle-related parameters, such as the muscular mass index, (MMI) ACT = 16.88 kg/m^2^ and SED = 14.66 kg/m^2^. Regarding physical activity parameters, energy expenditure and moderate to vigorous activity were significantly higher in the ACT group than in the SED. For a more detailed description, see [8].

### 3.2. Network Topology

**Figure 1:**
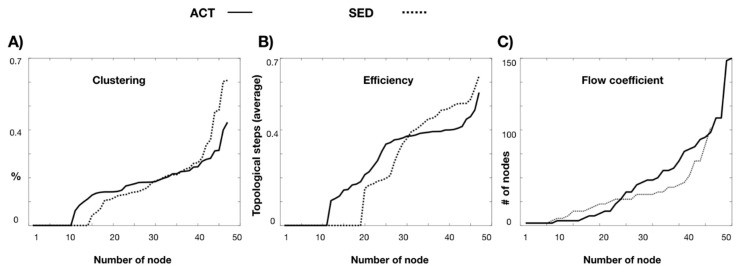
Topological comparison of active (ACT) and sedentary (SED) microbiota networks for clustering (**A**), efficiency (**B**), and flow coefficient (**C**). ACT group is represented with solid line and SED group with dotted line. # number.

The clustering distributions (Figure 1A) show that the bacterial microbiota of the SED network showed more bacteria with high clustering than the ACT network (a proportion of 0.37 and 0.22, respectively), although without statistical significance (*p*-corrected value = 0.63). The microbiota network global efficiency was statistically significantly higher (*p*-corrected value < 0.001) in the ACT network (a topological distance of 0.24 steps) than in the SED (an efficiency of 0.20) network (Figure 1B). When the flow coefficient was approached, it was significantly higher (*p* < 0.0001) in most of the bacteria in the ACT network than in the SED network, with mean values of 34 and 27.11 nodes for the ACT and SED networks, respectively (Figure 1C), indicating that the ACT network has a topology more suitable for information transmission or diffusion. Both the ACT and the SED networks have a positive assortativity coefficient, indicating that bacteria tend to link to other bacteria with the same or similar degree, and this link occurs significantly more frequently (*p* < 0.001) in the ACT network (a proportion of 0.19) than in SED network (a proportion of 0.08).

### 3.3. Conversion Networks

**Figure 2:**
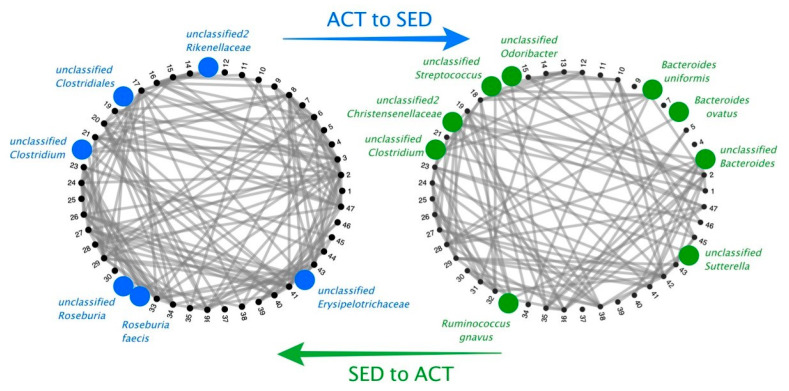
Network transition between ACT (blue) and SED (green) conditions, with the statistically significant (*p* < 0.001) driving keystone bacteria (represented by circles).

We considered the conversion of the ACT network to the SED network and vice versa. Figure 2 summarizes the driving keystone bacteria that are implicated in the reorganization or transition between ACT and SED conditions. Sorted in terms of flow coefficient relevance, the driving keystone microorganisms from the ACT to SED condition were: *Roseburia faecis*, an unclassified taxon from the *Roseburia* genus, an unclassified bacterial taxon from the Rikenellaceae family, an unclassified taxon from the Clostridiales order, and an unclassified bacterial taxa from the Erysipelotrichaceae family. The driving keystone bacterial taxa from the SED to ACT condition were as follows: an unclassified taxon from the *Sutterella* genus, an unclassified taxon from the *Bacteroides* genus (the *Bacteroides ovatus*, *Bacteroides uniformis*, and *Ruminococcus gnavus* species), an unclassified taxon from the *Streptococcus* genus, an unclassified taxon from the *Odoribacter* genus, an unclassified bacterial taxon from the Christensenellaceae family, and an unclassified taxon from the *Clostridium* genus.

## 4. Discussion

To understand the microbiota structure, as well as the bacterial taxa that play an important role within the microbiota structure, it is important to understand the relationship between the microbiota and its host interaction. Our analysis of the microbiota network did not identify the differentially abundant bacterial taxa but those that are key to the structure of the microbiota. This fact is important, since the pathogenicity of a microorganism is not always linked to its abundance, but can be linked to other factors, including interactions with other microbes and the host. However, from a network perspective, microbiota analysis is not yet widespread, although it was able to determine, for example, key changes in microbiota structure with pulmonary exacerbations in cystic fibrosis [13]. In our previous work, we determined that the microbiota network of sedentary individuals has reduced diversity and a less dense microbial network structure, with a lower density of connections and a lower level of competitive interactions [8]. In this work, we studied the topology of both ACT and SED microbiota networks. In terms of topology, the ACT gut microbiota network is more robust when considering the higher level of efficiency and the flow coefficient (capability of information transmission). We propose a method of relevant utility in microbiota studies. Our method supposes a novel procedure to analyze ecosystems based on the identification of driving keystone bacteria in a network reorganization that allows us to study the complexity of a process and opens the possibility to interact in different ways with the microorganism structure. The driving keystone bacteria, estimated here by the flow coefficient, are those with higher influence on other bacteria and with a higher capability to propagate their actions in the transition process between conditions. For these reasons, these driving bacteria are more suitable for interest in pharmacology or computational simulation studies than the identification of those with changes in levels of abundance. The driving keystone method could have implications for clinical studies, for example, in the neurodegenerative alterations found in Alzheimer’s disease or recovery from cancer treatment.

*Roseburia faecis* and an unclassified *Roseburia* species were two of the keystone bacteria with a higher degree of flow coefficient, and thus the most relevant in the reorganization that occurs when transitioning from an ACT to a SED network. This could partly explain why these species are key in the transition from an ACT to a SED network, as ACT subjects have a high fiber diet [8] and *Roseburia* species are short-chain fatty acid producers from non-fermentable carbohydrates that serve as a growth substrate [14], and can selectively promote key members of the microbiota [15]. *Roseburia* was also associated with a better physical condition [16] and occurs in a higher proportion of active women [17]. The presence of *Roseburia* is related to a more frequent intestinal transit, which could also explain why *Roseburia* species were representative of the ACT network, as ACT people usually have a greater intestinal transit [18], which could be a selective force on gut bacterial growth rates [19]. We cannot state this fact conclusively, however, as intestinal transit was not measured in our study. *Roseburia* is considered a health marker, as lower levels of this genus are associated with inflammatory disease, obesity, and diabetes [14,20,21]. According to these studies, our network analysis also shows that *Roseburia* is a key hub of a healthier and active lifestyle. Other key bacterial taxa in the ACT network are the *Rikenellaceae* and *Erysipelotrichaceae* families; however, the role of these bacterial taxa in human health is not yet clear. Although higher levels of the *Erysipelotrichaceae* family have been found in obese individuals [22], this butyrate-producer family is also associated with exercise and cardiorespiratory fitness [15,23]. These results may be contradictory; however, they both point out that the *Erysipelotrichaceae* family can be a key differentiating factor between individuals with ACT and SED lifestyles. In a study investigating the predictive variables of exercise in the gut microbiota, the *Rikenellaceae* family was shown to be a predictor of exercise, although its abundance was not modified by it [24]. This supports the idea that changes in the abundances of microbial taxa are not the only important factor, but also the role they play within the microbial ecosystem. On the other hand, an unclassified species of the *Sutterella* genus with a principal role in the transition from the SED to the ACT network was identified. At first, *Sutterella* was considered part of the commensal microbiota without playing a major role in it; however, in recent years, the presence of *Sutterella* has been associated with neurological disorders such as multiple sclerosis and autism [25,26], and with conditions such as ulcerative colitis, in which it impairs the immune system’s function [27], and has been identified as a common driver taxon in diabetes [28]. *Ruminococcus gnavus* is a mucin-degrader of the intestine, producing a polysaccharide with pro-inflammatory activity [29], and its presence was found to be increased in inflammatory bowel diseases [29,30]. The increased presence of this bacterium in intestinal diseases may be derived from its resistance to oxidative stress. This may also be the reason why it is found in greater abundance in people who exercise, since exercise is associated with higher levels of oxidative stress [31]. Our microbiota network topology analysis reveals that the microbiota of the ACT group is a microbiota with higher overall efficiency and greater transmissibility. The key bacteria linked to the transition from a sedentary to an active lifestyle and vice versa involves bacteria related to health and disease. These results suggest that the transition from an active to a sedentary lifestyle leads to changes in health-promoting key taxa of the network, and the transition from SED to ACT can lead to the modification of key bacteria related to diseases. It should be noted that both groups of participants were healthy people, which would indicate that in the microbial network of healthy sedentary people, disease-related bacteria can be identified and could be proposed as early markers of disease. The topological study of the microbial network is a new approach to the study of the microbiota, which can provide important new parameters in the interaction between the different microorganisms and with the host.
